# Micropipette aspiration reveals differential RNA-dependent viscoelasticity of nucleolar subcompartments

**DOI:** 10.1073/pnas.2407423122

**Published:** 2025-05-28

**Authors:** Holly H. Cheng, James V. Roggeveen, Huan Wang, Howard A. Stone, Zheng Shi, Clifford P. Brangwynne

**Affiliations:** ^a^Department of Molecular Biology, Princeton University, Princeton, NJ 08544; ^b^Department of Mechanical and Aerospace Engineering, Princeton, NJ 08544; ^c^Department of Chemistry and Chemical Biology, Rutgers University, Piscataway, NJ 08854; ^d^Princeton Materials Institute, Princeton University, Princeton, NJ 08544; ^e^Department of Chemical and Biological Engineering, Princeton University, Princeton, NJ 08544; ^f^Omenn-Darling Bioengineering Institute, Princeton University, Princeton NJ 08544; ^g^HHMI, Chevy Chase, MD 21044

**Keywords:** biomolecular condensates, rheology, soft matter, nucleolus, organelles

## Abstract

The nucleolus is a multiphasic biomolecular condensate that facilitates ribosome biogenesis, a complex process involving hundreds of proteins and RNAs. The proper execution of ribosome biogenesis likely depends on the material properties of the nucleolus. However, these material properties remain poorly understood due to the challenges of in vivo measurements. Here, we use micropipette aspiration (MPA) to directly characterize the viscoelasticity and interfacial tensions of nucleoli within transcriptionally active *Xenopus laevis* oocytes. We examine the major nucleolar subphases, the outer granular component (GC) and the inner dense fibrillar component (DFC), which itself contains a third small phase known as the fibrillar center (FC). We show that the behavior of the GC is more liquid-like, while the behavior of the DFC/FC is consistent with that of a partially viscoelastic solid. To determine the role of ribosomal RNA in nucleolar material properties, we degrade RNA using RNase A, which causes the DFC/FC to become more fluid-like and alters interfacial tension. Together, our findings suggest that RNA underlies the partially solid-like properties of the DFC/FC and provide insights into how material properties of nucleoli in a near-native environment are related to their RNA-dependent function.

## Significance

Biological reactions are strongly coupled to the structure and properties of their environment. Cellular compartmentalization into biomolecular condensates with distinct material properties enables a diverse array of biological processes. However, the link between material properties and function is challenging to study due to the difficulties of measuring intracellular condensate material properties. Here, we probe the material properties of a biochemically active condensate, the nucleolus, inside a living cell nucleus using micropipette aspiration (MPA). We find that the nucleolus is more solid-like at its core and more liquid-like toward its periphery. These properties are RNA-dependent; by degrading RNA, the nucleolar core becomes more liquid-like. These results provide insight into how nucleolar material properties may be linked to the sequential steps of ribosome maturation.

The interior of living cells is compartmentalized into both membrane-bound and membrane-less organelles. Many membrane-less organelles, from nucleoli and Cajal bodies in the nucleus to P-bodies and stress granules in the cytoplasm, form through phase separation and related phase transitions, with the resulting condensed assemblies of proteins and RNAs referred to as biomolecular condensates (1–3). The kinetic and thermodynamic driving forces underlying condensate formation can give rise to a wide range of material states, from liquid-like to gel or solid-like. These condensate material states are correlated with a variety of biological functions (4–9), and aberrant changes in condensate material state are associated with biological dysfunction and disease. For example, condensate liquid-to-solid transitions are linked to various neurodegenerative diseases (10, 11), including those driven by FUS (12), tau (13), polyQ (14), alpha-synuclein (15), hnRNPA1 (16), and other proteins.

Condensate material states can be characterized in terms of macroscopic material properties, which include viscosity, elasticity, and interfacial tension (17, 18). A major challenge in the condensate field is the difficulty of in vivo measurements of these material properties. While many rheological techniques are capable of measuring such properties with in vitro condensates, these reconstituted systems are vast simplifications of native condensates in their cellular context. Existing methods for inferring condensate material properties in vivo rely on indirect measurements and often use condensates containing overexpressed, fluorescently tagged proteins, which can give rise to altered material properties. Within living cells, condensate viscosity has been very roughly estimated from measurements of the diffusion coefficient of fluorescently labeled condensate proteins using fluorescence recovery after photobleaching (FRAP) or fluorescence correlation spectroscopy (FCS) (19–21). However, the diffusion of one protein species may not fully reflect the bulk rheology of the whole condensate, which could contain hundreds of different protein species, and assumptions of molecular size, shape, and material homogeneity are needed to convert the measured diffusion coefficient to condensate viscosity.

Another material property of interest is interfacial tension (22), which in principle can be estimated from the fluctuations in droplet shape (23), but for condensates also typically requires fluorescently labeled proteins, and is challenging due to the small scale of typical fluctuations (24–26). The relaxation time of coalescing condensates has also been used to approximate the ratio between viscosity and interfacial tension (inverse capillary velocity) (24, 27), but must be paired with another technique to extract viscosity, which can usually only be estimated roughly. An additional challenge is that these techniques require assuming that condensates are liquid-like, Newtonian fluids (28), even while many condensates appear to exhibit characteristics of gel- or solid-like materials. For instance, condensates with proteins that exhibit incomplete FRAP recovery (8, 9), or those that do not dissolve following salt/chemical treatment (29, 30), are thought to be more solid-like, although these techniques only provide qualitative observations.

Given the limitations of current techniques used to characterize the viscoelastic and surface properties of condensates in cells, there is a need for approaches that can directly measure in vivo condensate material properties. Micropipette aspiration (MPA) is a well-established, label-free technique for probing the mechanical properties of cells (31, 32), and has recently been applied to study the viscoelasticity and interfacial tension of in vitro condensates (33–37). MPA measures a one-dimensional strain in response to an applied stress, which takes the form of the pressure drop imposed across the pipette. The minimum pressure required for a condensate to enter the pipette is a direct measure of interfacial tension (34, 35, 38). Once inside the pipette, generally, a fluid-like, Newtonian material will deform with a constant strain rate $\dot{\varepsilon }$ under applied constant stress, σ, i.e., $\sigma =\eta \dot{\varepsilon }$, while a solid-like material deforms with a constant strain, ε, i.e., σ=Eε, where η and E are, respectively, the viscosity and elastic modulus of the material. Viscoelastic materials display strain responses consistent with both fluid-like and solid-like behaviors at short times, but may generally be classified into fluid-like and solid-like based on their long-time behavior. Because MPA requires condensates larger than the micropipette opening diameter, adapting MPA for studying intracellular condensates, especially those inside the cell nucleus, is challenging due to the small size of typical condensates (39).

To leverage the full power of MPA for measuring condensates in cells, we study the large nuclear condensates within *Xenopus laevis* oocytes, which are structurally and compositionally similar to those in typical mammalian systems (27, 40, 41). The potential for deploying MPA and related techniques for measuring native condensate properties in *X. laevis* oocytes is particularly attractive for the nucleolus — the largest and still elusive multiphasic biomolecular condensate responsible for the production of ribosomes, highly complex assemblies composed of ~80 proteins and four ribosomal RNAs (rRNAs) in eukaryotes (42). Three rRNAs are transcribed within the nucleolus, where they are modified, and assembled with dozens of proteins into precursor ribosomal subunits. These processing steps are spatially organized within the nucleolus in three concentrically arranged subcompartments, with the fibrillar center (FC) as the innermost compartment, followed by the dense fibrillar component (DFC) and granular component (GC). The nucleolus exhibits liquid-like material behaviors (24, 27), and the proper execution of ribosome biogenesis may be coupled to these properties. For example, inducing nucleolar gelation through oligomerization of the GC protein, nucleophosmin (NPM1), leads to the accumulation and depletion of different precursor rRNAs (4). Moreover, the transcription and processing of rRNA may modulate the material properties of the individual phases of the nucleolus. Indeed, newly transcribed rRNAs in the DFC have been proposed to entangle and form a gel-like state, while rRNAs that have been packaged and assembled into preribosomal subunits in the GC could contribute to a more liquid-like state (43). The potential for significant RNA-dependent viscoelasticity in the nucleolus would be consistent with previous measurements on the incomplete FRAP recovery of FIB1-eGFP (44). However, the lack of direct measurements of nucleolar material properties severely limits our understanding of these properties and the potential contributions of rRNAs.

Here, we use MPA to directly characterize the viscoelastic properties and interfacial tensions of nucleoli within transcriptionally active *X. laevis* oocytes and elucidate the role of RNA in influencing these material properties. We show that the major nucleolar compartments, the GC and DFC/FC, have distinct material responses. Specifically, the GC is liquid-like, while the DFC/FC is more solid-like. We further show that these differences arise largely from RNA, whose degradation fluidizes the DFC.

## Results

### Fluid Behaviors of Nucleoli During MPA.

**Fig. 1.**
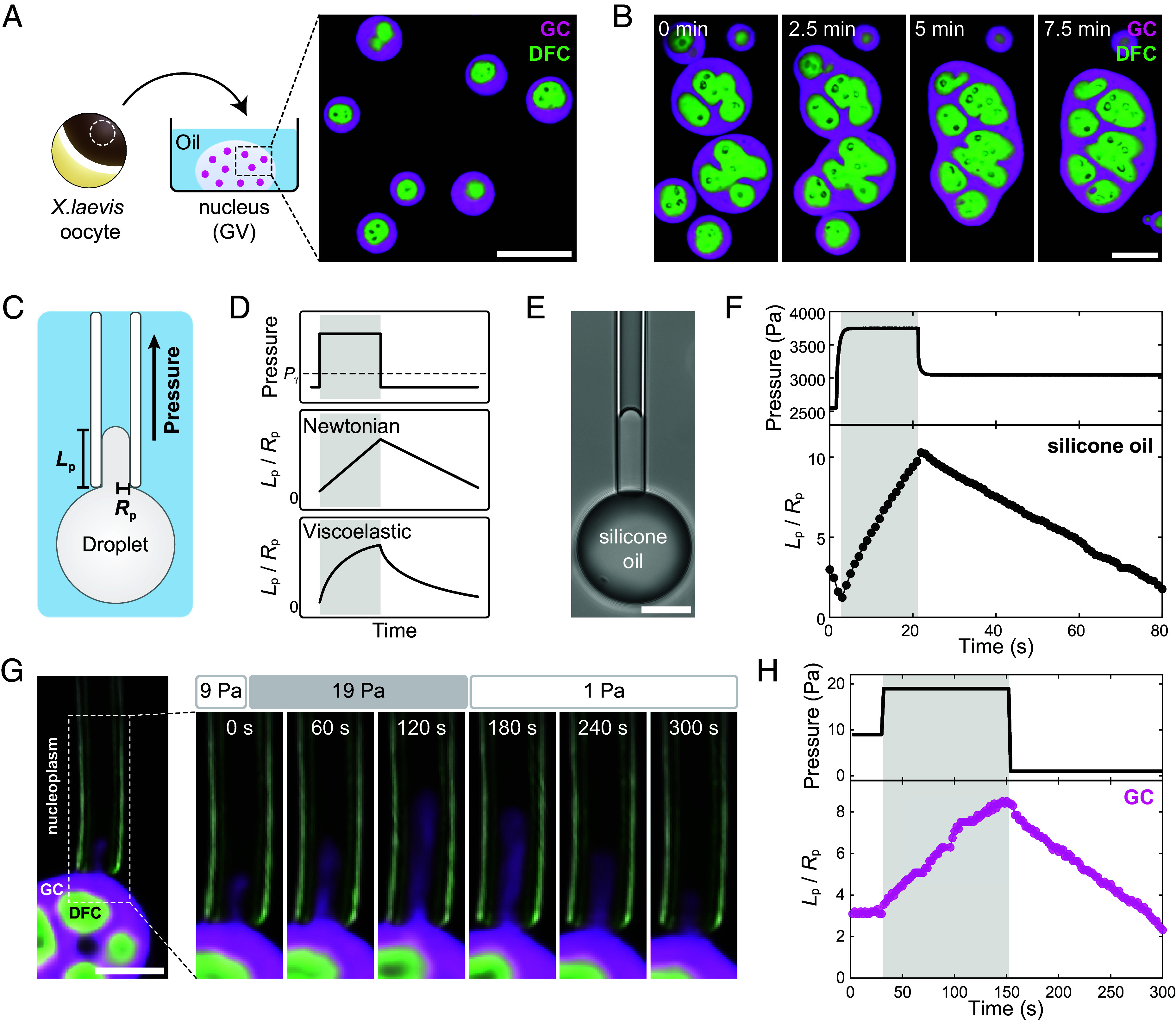
Liquid-like behaviors of nucleoli when aspirated into a micropipette. (*A*) Schematic of the experimental setup. The Stage V-VI *X. laevis* oocyte nucleus (GV) is isolated into mineral oil to facilitate imaging. The image shows a multicolor confocal Z-slice through the bottom of a GV. In the nucleoli, the GC is labeled with NPM1-RFP (magenta), and the DFC is labeled with FIB1-eGFP (green). (Scale bar, 15 µm.) (*B*) Timelapse of nucleoli fusing after disruption of the surrounding actin network with Latrunculin A. (Scale bar, 10 µm.) (*C*) Schematic of MPA where a nonwetting droplet is aspirated into a pipette. Abbreviations: *L*_p_ = length of the droplet inside the pipette. *R*_p_ = radius of the pipette. Positive aspiration pressure leads to increasing *L*_p_. (*D*) Expected mechanical strains of Newtonian and viscoelastic fluids after a constant stress (pressure) is applied and released. For the Newtonian fluid, we expect a linear response, whereas for a viscoelastic fluid, we expect a nonlinear response. As an example of a viscoelastic fluid, we plot the results from the Kelvin-Voigt model. $P_{\gamma }$ represents the capillary pressure that must be exceeded by applied external pressure to induce the flow of condensate into the micropipette. (*E*) Brightfield image of a silicone oil droplet partially aspirated into a micropipette. (Scale bar, 20 µm.) (*F*) A representative example of the silicone oil aspiration response. (*G*) Multicolor confocal timelapse images of the GC entering the micropipette under suction pressure overlaid with simultaneously acquired differential interference microscopy (DIC) images. The micropipette appears slightly green due to adsorption of nucleoplasmic FIB1-eGFP. (Scale bar, 5 µm.) (*H*) Quantification of the GC deformation shown in panel *G*.

To probe the material properties of the nucleolus, we take advantage of the large ~10 μm nucleoli of stage V-VI *X. laevis* oocytes, which are single cells that reach diameters of 1 to 1.3 mm. The nucleus of each oocyte, known as the germinal vesicle (GV), contains 1,400 to 1,600 nucleoli that form around extrachromosomal rDNA repeats and are suspended within an actin meshwork (45–47). *X. laevis* nucleoli share a similar tripartite organization with mammalian cells, containing nested FC, DFC, and GC compartments. While nucleoli can be visualized without fluorescent labeling by differential interference contrast (DIC) microscopy, to distinguish the different nucleolar subcompartments, we label the GC and DFC by expressing fluorescently tagged nucleophosmin (NPM1-RFP) and fibrillarin (FIB1-eGFP), respectively. Because the oocyte cytoplasm is opaque, we isolate the GVs into mineral oil to facilitate imaging (Fig. 1*A*). As previously described, these nucleoli exhibit liquid-like behaviors (24, 27, 44), and disrupting the actin meshwork causes sedimentation under gravity and apparently liquid-like fusion of the GC and DFC (Fig. 1*B*) (47).

Late stage *X. laevis* oocyte nucleoli are known to be transcriptionally active (48). To independently confirm this activity, we inject the oocytes with 5-ethynyl uridine (5-EU), which is incorporated into nascent RNA transcripts. By incubating the injected oocytes for increasing times, then fixing the nucleoli and conjugating Alexa Fluor 647 to 5-EU, we can visualize the movement of fluorescently labeled nascent transcripts over several hours (*SI Appendix*, Fig. S1*A*). We observe a 5-EU signal higher than the background inside the nucleoli (*SI Appendix*, Fig. S1*B*). Moreover, when the oocytes are incubated with 5-EU for longer, the 5-EU signal spreads from the DFC to the GC, similar to prior observations in actively transcribing mammalian cells (43). These observations suggest that 5-EU is incorporated into nascent rRNAs, confirming that there is active transcription within these nucleoli. Additionally, these nucleoli remain transcriptionally active following isolation of the GV into oil (49). To confirm this activity, we injected 5-EU at increasing times following GV isolation. We observe that in GVs injected two hours post isolation, 5-EU signal is present but significantly lower than GVs injected immediately following isolation (SI Appendix, Fig. S1 *C*–*D*). These results indicate that the nucleoli are transcriptionally active for at least two hours after GV isolation, but exhibit notable decline in activity over time; here, we complete all experiments within two hours of GV isolation.

Before applying MPA to nucleoli, we first consider the expected aspiration responses for droplets with Newtonian and viscoelastic material properties. In our MPA experiments, we use a micropipette to aspirate a droplet and determine the relationship between the pressure applied and the corresponding deformation length of the droplet into the pipette (*L*_p_) (Fig. 1*C*); aspiration is characterized as positive suction pressure and leads to increasing *L*_p_. For a Newtonian fluid that wets the pipette, the relationship between *L*_p_ versus time should be approximately that of a square root, *L*_p_ ~ $\sqrt{t}$ (36). However, for a Newtonian fluid that does not wet the glass, we expect that there will be a film of solvent that lubricates the aspirant (50). The lubrication effect leads to a near-linear relationship between *L*_p_ and time (Fig. 1*D*), as observed for silicone oil under such conditions (Fig. 1 *E*-*F*). In contrast, we expect that the evolution of *L*_p_ for a nonwetting viscoelastic fluid is nonlinear in time (Fig. 1*D*).

We now measure nucleolar material properties, leveraging the accessibility of nucleoli within the extracted GV. After inserting the pipette initially filled with a buffer into the GV and bringing it into proximity with the nucleolar surface, we apply suction pressure (19 Pa), causing the nucleoplasm, followed by the nucleolus, to flow into the pipette (Fig. 1*G* and Movie S1). Initially, only the outer GC nucleolar compartment enters the pipette. We observe that the curvature of the GC within the pipette is consistent with the expectation for a nonwetting fluid. To further verify the presence of a thin layer of nucleoplasm between the nucleolus and pipette wall, we fluorescently label the nucleoplasm using a 150 kDa FITC-dextran, which is excluded from the nucleolus (*SI Appendix*, Fig. S2*A*) (40). As expected, the fluorescently labeled nucleoplasm can be seen clearly as a thin layer between the GC and pipette wall (*SI Appendix*, Fig. S2*B**)*. For both the GC and silicone oil under nonwetting conditions, we also observe necking instabilities, reminiscent of the Rayleigh–Plateau instability (51), which lead to small droplets pinching off inside the pipette (*SI Appendix*, Fig. S2 *C-D*). The presence of these instabilities, together with our other observations, indicate that the nucleolus is aspirated into a nonwetting cylinder inside the micropipette. Moreover, similar to silicone oil, the time evolution of *L*_p_ for the GC under aspiration conditions is nearly linear (Fig. 1*H*). This response suggests that the GC exhibits fluid-like, approximately Newtonian properties on long, > 10 s, timescales.

### The Nucleolar Subcompartments have Distinct Material Properties.

**Fig. 2.**
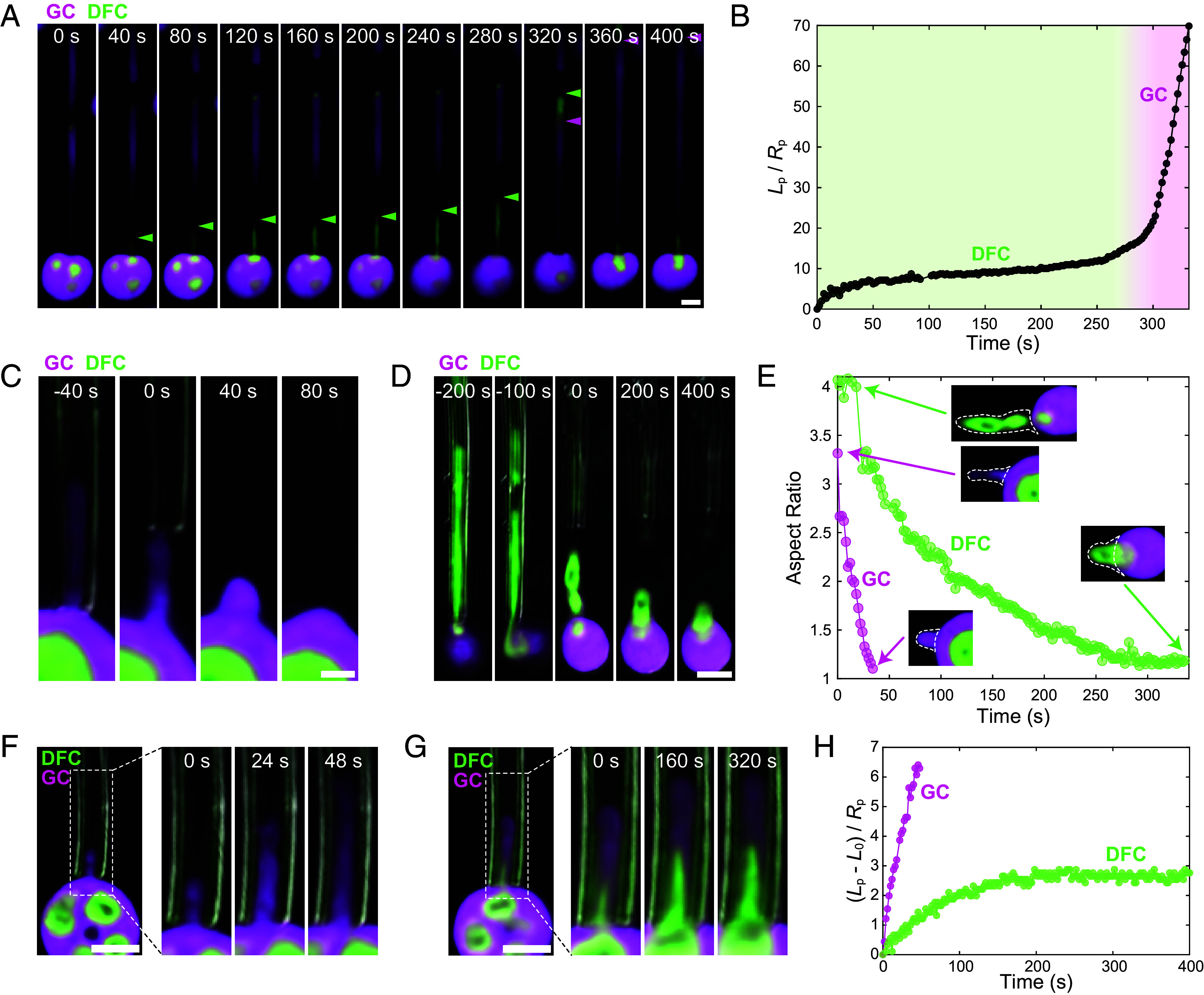
Nucleolar subcompartments have distinct material properties. (*A*) Fluorescence confocal timelapse images of a nucleolus aspirated under constant pressure (360 Pa). The green arrowheads point to the tip of the DFC inside the pipette, while the magenta arrowheads point at the beginning of the GC phase. (Scale bar, 5 µm.) (*B*) Plot of the lead position of the DFC (indicated by the green arrowhead in panel *A*) over time. (*C*) Fluorescence confocal timelapse images, overlaid with DIC images, showing the relaxation of a cylindrical plug of GC, generated by ejecting the aspirated nucleolus (negative pressure) while pulling the pipette away. (Scale bar, 2 µm.) (*D*) Ejection of the aspirated GC and DFC shows slow relaxation of the DFC. (Scale bar, 5 µm.) (*E*) Aspect ratio of the relaxing cylindrical plug of GC and DFC shown in panels C and D over time. The white outlines in the insets indicate the regions of the relaxing nucleolus included in the analysis. The time when the nucleolus fully exits the pipette is defined as time = 0. (*F*–*H*) Aspiration of the GC and DFC of the same nucleolus at 20 Pa. *L*_0_ = length of the droplet inside the pipette at time = 0. (Scale bar, 5 µm.)

Under constant suction pressure, the internal DFC subcompartment of nucleoli can also be aspirated into the pipette, allowing us to probe its properties as well; we note that aspiration of the DFC almost always entails simultaneous aspiration of the FC within, but for simplicity we refer to this is a single composite DFC (see further discussion below). When the DFC is larger than the pipette opening, the aspiration is significantly slowed, suggesting that the DFC material resists deformation more than the GC (Fig. 2 *A* and *B* and Movie S2); when the DFC is fully deformed and aspirated into a cylindrical shape within the pipette, the aspiration rate can be seen to dramatically increase (Fig. 2 *A* and *B* and Movie S2). To further study the qualitative aspiration behaviors of the DFC compared with the GC, we next examine the relaxation after an aspirated nucleolus is ejected (applying negative pressure). The GC that is ejected without an enclosed portion of DFC quickly returns to a nearly spherical shape as it leaves the pipette, as expected from the effects of interfacial tension in rounding a simple liquid droplet (*SI Appendix*, Fig. S3). In these conditions, GC relaxation is faster than the ejection time, making it challenging to determine the shape relaxation timescale. Therefore, we generated a more fully cylindrical initial condition by rapidly pulling away the pipette from the ejecting nucleolus; this initially cylindrical plug of GC fully rounds up in less than 60 seconds (Fig. 2*C* and Movie S3). In contrast to the GC, the ejected DFC and its associated film of surrounding GC typically retain a cylindrical shape for much longer, > 200 seconds in the example shown in Fig. 2*D* and Movie S4; the relaxation of the cylindrical plug of the GC versus DFC outside the pipette are plotted in Fig. 2*E*. These results provide additional evidence that the GC and DFC have different material properties, consistent with the DFC exhibiting significantly slowed internal relaxation dynamics. Moreover, these results suggest that the slow relaxation time of the entire nucleolus (e.g., Fig. 1*B*) may be governed by the slow internal rearrangement of the DFC.

### The Nucleolar Granular Compartment is a Newtonian Liquid-Like Material, while the DFC Exhibits Behavior Consistent with a Viscoelastic Solid-Like Material.

To further investigate the apparent differences in material properties of the GC and DFC, we perform constant pressure creep aspiration tests of GC and DFC in the manner previously described (34, 36). Specifically, our experiments consist of a single step change in the applied pressure, driving material aspiration, followed by the applied pressure being turned off, leading to relaxation driven by interfacial tension. In all these experiments, the nucleolus starts partially aspirated into the pipette; doing so greatly simplifies our later analysis, as the dynamics of a droplet initially entering a pipette can be difficult to interpret. We apply relatively small suction pressures (typically <50 Pa) to ensure that the nucleolar subcompartments always remains partially unaspirated, reducing the potential contributions from changing the nucleolar geometry outside the pipette over time. An example of the creep aspiration test performed on the GC and DFC of the same nucleolus at the same suction pressure (20 Pa) is displayed in Fig. 2 *F*–*H*. When we aspirate the GC by itself and track the change in aspiration length (Fig. 2*F*), on the timescales of our experiments (>~10 s), the curve of *L*_p_ versus t for GC shows a nearly linear relationship, consistent with the GC being Newtonian, liquid-like, and deformable with a roughly constant strain rate under a constant stress. When both the GC and DFC are aspirated into the pipette, with DFC at the pipette tip and GC forming a plug in the pipette, we expect that the DFC controls the aspiration response and that the GC plug is advected with the bulk of the DFC (*SI Appendix*, Fig. S4). While the GC plug will change the effective pressure difference experienced by the DFC (36), knowing the exact pressure drop acting on the material requires knowledge of both the plug effects and material’s interfacial tensions, but these effects are expected to be small at the applied suction pressures (*SI Appendix*, Text). We observe that the DFC initially flows into the pipette (Fig. 2*G* and Movie S5), before settling to a nearly constant position that stays fixed for long times. This behavior of the DFC at these long time-scales is consistent with that of a viscoelastic solid, further supporting the qualitative conclusions drawn in the previous section.

**Fig. 3.**
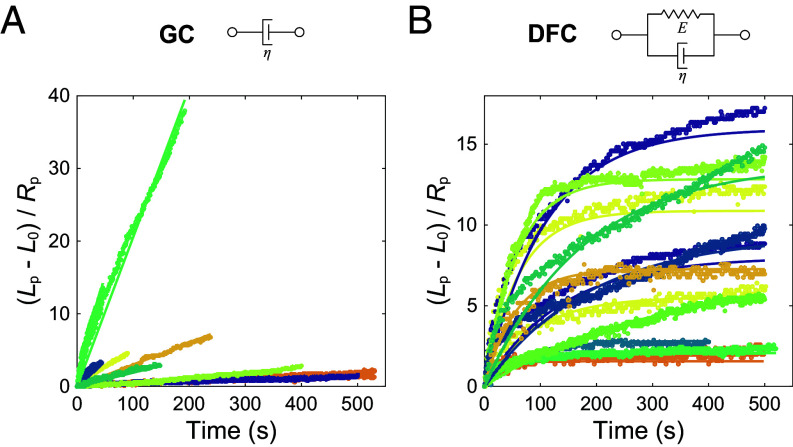
Comparison of the material properties of GC and DFC. (*A* and *B*) Aspiration responses of the GC (*A*) and DFC (*B*). The dots show the experimental data for the strain of the GC aspirated at 5 Pa (n = 14 measurements from 8 nucleoli) and DFC aspirated at 20 Pa (n = 13 measurements from 10 nucleoli). The lines represent the results of fitting Eq. **1** to the experimental data. Each color represents measurements taken using different nucleoli.

For a particular pressure (P = 5 Pa for the GC and *P* = 20 Pa for the DFC), we repeatedly aspirate the GC and DFC across different samples, consistently revealing this nearly linear aspiration response for the GC and nonlinear response for the DFC curves, despite significant variability across samples (Fig. 3); these qualitative features of the GC versus DFC stress response hold across a range of applied suction pressures and are independent of the overexpression of fluorescent GC and DFC proteins, and the time since the GV was isolated (*SI Appendix*, Figs. S5–S7). We only further analyzed measurements where there was no visible necking of the GC and DFC within the pipette, as necking instabilities make the aspiration response curves difficult to interpret (*SI Appendix*, Fig. S8). Our full dataset includes measurements taken using oocytes from multiple animals combined over several experimental conditions (NPM1-RFP/FIB1-eGFP expression level and time since GV isolation) for a total of 14 measurements taken from 8 nucleoli for the GC, and 13 measurements taken from 10 nucleoli for the DFC.

We next carefully examine the stress–strain behavior of aspirated GC and DFC to better characterize the viscoelastic response of each. The constant pressure creep aspiration experiments measure one-dimensional strain as a function of the applied aspiration pressure, allowing us to characterize the aspiration responses in terms of one-dimensional spring-damper models (*SI Appendix*, Fig. S9 and Table S1). The fitted spring-damper coefficients are related to the fundamental properties of the material (e.g., storage G′ and loss G″ moduli). However, precisely connecting these requires analysis of the full three-dimensional flow problem. Even for a Newtonian fluid, the effective viscosity is a function of the Newtonian shear viscosity and the flow geometry (36, 50). Nonetheless, the fitting parameters themselves can reveal the approximate order of magnitude of the more fundamental constants (32, 52).

We define a strain ε = (*L*_p_ – *L*_0_)/*R*_p_, where *L*_0_ is *L*_p_ at the start of aspiration and *R*_p_ is the radius of the pipette opening. In the case of a constant applied pressure *P*, the actual stress σ experienced by the material will be a combination of the applied pressure and a Laplace pressure drop $P_{\gamma }$, i.e. $\sigma =P-P_{\gamma }$. The relationship between the Laplace pressure to the interfacial tension γ is $P_{\gamma }$ = 2 γ (1/*R*_p_ - 1/*R*_c_), where *R*_c_ is the radius of the droplet outside of the pipette. Following previously published methods (34, 36), we estimate the GC-nucleoplasm interfacial tension to be 1.7 ± 0.3 µN/m (*SI Appendix*, Fig. S10). This estimate is on the same order of magnitude, approximately 4-fold larger, as a previously reported indirect estimate for *X. laevis* oocyte nucleolar interfacial tension determined by another method (0.4 ± 0.1 µN/m) (44). While it is difficult to directly measure the DFC–GC interfacial tension, we can assign an upper bound interfacial tension of around 0.5 ± 0.3 µN/m (*SI Appendix*, Text). The error values of the parameters are reported as one SEM. For more discussion of error estimates, see *Methods*.

For spring/damper models with up to two-elements, the general form of the strain response to a constant stress σ is given by

[1] $$\varepsilon (t)=A+Bt+Ce^{-Dt},$$

where the constants for different models are given in *SI Appendix*, Table S1. While there are many examples of more complicated viscoelastic models constructed by combining more springs and dampers, we will focus only on the two simplest examples of a viscoelastic fluid (Maxwell) and viscoelastic solid (Kelvin-Voigt). Adding more components would allow for additional degrees of freedom when fitting the data, and thus reduce mean-squared error (MSE); for example, adding a third component can reduce the MSE in fitting the DFC response by a factor of three. However, this is unlikely to yield additional insight, and for the sake of simplicity we choose to focus only on two-element models to highlight the most salient features of the material response. The case B=0 and C≠0 corresponds to two-element solid-like models (those that decay to a constant strain) while B≠0 and C=0 corresponds to two-element fluid-like models. We neglect Hookean models, as no aspiration curves show evidence of pure elastic behavior. Also, note that since by definition σ depends on the unknown interfacial tension γ, each model has one additional unknown material parameter related to the interfacial tension.

We split the time series data for the GC and DFC into an aspiration (Fig. 3) and relaxation segment (*SI Appendix*, Fig. S11), corresponding to positive and negative rates of strain. Here, we analyze only the aspiration segments, as there are caveats associated with the relaxation dynamics upon removal of positive suction pressure (*SI Appendix*, Text), and the pressure-driven aspiration data are sufficient for determining solid-like versus liquid-like behavior of the two materials. In fitting the GC data, the best numerical performance is achieved by setting C=0, which is characteristic of either a Newtonian- or Maxwell-like response, again consistent with a more fluid-like GC (*SI Appendix*, Text). The Maxwell model yields unphysical values for the elasticity (*SI Appendix*, Text), and thus we focus on the Newtonian model for the GC, which corresponds to the generally linear shape of GC aspiration data, as shown in Fig. 3*A*.

In considering the DFC aspiration data, within the family of two-element models, the minimum error and best numerical stability (as determined by sensitivity to the initial parameters) is achieved by assuming a solid model (Kelvin-Voigt) and fixing B=0. Since we normalize initial strain to zero, A=-C. The resulting fit reduces the mean squared error when compared to a Newtonian model (C=D=0) by over 6.5 times, at the cost of one additional fitting parameter representing elastic effects. This provides evidence that DFC behaves more like a viscoelastic solid than a fluid, which is consistent with the curved, exponential decay shape of DFC aspiration data, as shown in Fig. 3*B*.

We can use this aspiration data to estimate values for viscoelastic properties of the two materials. We can interpret the fitting parameters in terms of the definitions given in *SI Appendix*, Table S1. Applying this method to our GC experiments, we find an average GC viscosity of 220 ± 100 Pa∙s. Combining this value with our estimate of interfacial tension (1.7 ± 0.3 µN/m) gives an inverse capillary velocity (η/γ) of approximately 130 ± 60 s/µm, which is approximately 4 times larger than a previous estimate (30 ± 5 s/µm) based on fusion analysis (44). However, it is important to note that the measured viscosity appears to vary by over two orders of magnitude in our experiments. This may be due to inherent biological variability, but is likely also impacted by the statistically significant correlation between the GC viscosity and the time since the GV was isolated, potentially resulting from the slow decrease in metabolic activity after isolation (*SI Appendix*, Fig. S7 *A* and *C*). Indeed, if we discard all samples that had been isolated for over one hour, the mean viscosity falls to 38 ± 10 Pa∙s, resulting in an inverse capillary velocity of 22 ± 7 s/µm, which is in line with previous estimates.

Turning now to the DFC, we first note that the GC–DFC interfacial tension is small (~0.5 ± 0.3 µN/m), and so the Laplace pressure it generates is also small relative to the applied pressure of 20 Pa (*SI Appendix*, Text). If we only consider the effects of GC-nucleoplasm interfacial tension while ignoring GC–DFC interfacial tension, we interpret the fitting parameters as indicating an effective DFC viscosity of 250 ± 60 Pa∙s and DFC elastic modulus of 3.1 ± 0.8 Pa. Accounting for the effects of the GC–DFC interfacial tension using our estimated upper bound minimally affects the fitting parameters; we find an effective DFC viscosity of 210 ± 50 Pa∙s and an effective DFC elastic modulus of approximately 2.6 ± 0.7 Pa. We observe a moderate correlation between DFC properties and time since GV isolation, but this effect is not statistically significant (*SI Appendix*, Fig. S7 *B*, *D*, and *E*). Also, we do not observe a statistically significant effect of fluorescent labeling of the GC and DFC on the measured material properties (*SI Appendix*, Fig. S7 *C*–*E*); however, it is possible that there are effects of labeling when comparing measurements made at similar times following GV isolation that would require a larger sample size to capture. While our results in the case of the GC are close to previously published estimates, our measured values should be treated as approximations (*Methods*), due to these potential impacts of fluorescent labeling and time since isolation, as well as the impact of geometric and flow factors.

### Degradation of RNAs Fluidizes the DFC.

**Fig. 4.**
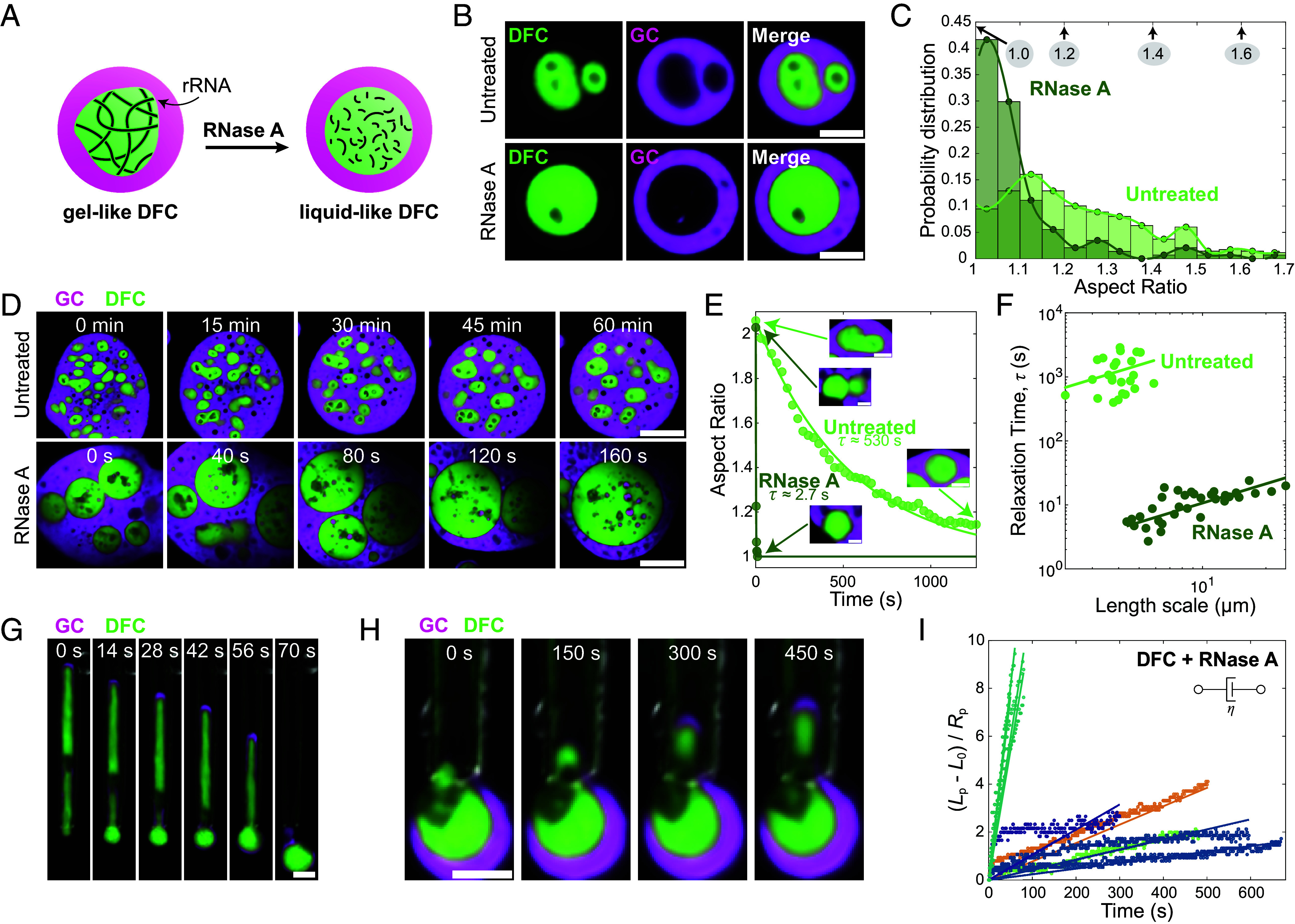
Degradation of RNAs fluidizes the DFC. (*A*) Schematic showing the predicted effect of RNase A. (*B*) Representative images of untreated and RNase-treated nucleoli. (Scale bar, 5 µm.) (*C*) DFC aspect ratio probability distribution histograms for untreated (n = 366 DFCs) and RNase A treated (n = 147 DFCs) nucleoli. The average aspect ratio of untreated DFCs is 1.28 ± 0.02 (mean ± SEM), and for RNase-treated DFCs is 1.10 ± 0.01. (*D*) Fusion of untreated and RNase-treated nucleoli induced by inhibiting actin polymerization using Latrunculin A. (Scale bar, 15 µm.) (*E*) Single example fusion events of untreated (light green curve) and RNase-treated (dark green curve) DFCs of comparable sizes. The solid lines represent exponential fits to the data. The inset shows the DFCs before and after fusion. (Scale bar, 3 µm.) (*F*) DFC fusion relaxation times, τ, versus length, *L*, for untreated (n = 24 DFCs) and RNase-treated (n = 34 DFCs) nucleoli. (*G*) Ejection of an RNase-treated DFC shows rapid relaxation. Fluorescent images are overlaid with DIC images to help visualize the micropipette. (Scale bar, 3 µm.) (*H*) Aspiration of an RNase-treated nucleolus. (Scale bar, 3 µm.) (*I*) Aspiration responses of RNase-treated DFCs at 20 Pa exhibit more linear behavior compared with untreated conditions (Fig. 3*B*). The dots represent experimental data and the lines show the fit to the Newtonian model. Each color represents a different nucleolus, n = 9 measurements from 5 nucleoli.

We reasoned that the material properties of nucleolar subcompartments, particularly the DFC, could be dominated by the high concentrations of newly transcribed ribosomal RNA, as suggested in recent work (43). If the nascent ribosomal RNAs are transcribed at the center of the nucleolus at a high enough concentration, they could form a partially entangled, gel-like state, potentially contributing to the observed viscoelastic solid signature of the DFC (Fig. 3*B*). To test this, we inject RNase A into the GV, which degrades RNAs and thus should fluidize the DFCs (Fig. 4*A*). Consistent with this physical picture, following the degradation of RNAs, we observe that the DFCs of nucleoli rapidly coalesce and become more spherical (Fig. 4 *B* and *C*). We also observe a striking increase in the fusion rate of the RNase-treated nucleoli, with the DFCs of nucleoli fusing in seconds, as compared to DFCs in untreated GVs, which typically fuse more slowly (Fig. 4 *D* and *F* and Movies S6 and S7). Remarkably, the inverse capillary velocity (η/γ) of RNase-treated nucleolar DFCs (~1 s/µm) is two orders of magnitude smaller than in untreated nucleoli (~300 s/µm). Further suggestive of changing material properties, we observe that the aspirated RNase-treated DFCs immediately return to a spherical shape upon ejection from the pipette (Fig. 4*G* and Movie S8). This RNase-treated DFC behavior is similar to the untreated GC and unlike the untreated DFCs, which retain a cylindrical shape outside the pipette for several minutes (Fig. 2*D*). These observations are consistent with RNA degradation causing dramatically decreased viscosity and/or increased DFC–GC interfacial tension; however, observations of DFC relaxation alone are unable to disentangle these two possible effects.

To determine the relative contributions of potential RNA-dependent viscosity and interfacial tension to the nucleolar material properties, we quantitatively examined the changes in RNase-treated DFC material properties using MPA. As before, we focus primarily on deformation upon aspiration into the micropipette (Fig. 4*H* and Movie S9). While in untreated DFCs, we observe a nonlinear aspiration response with the deformation saturating at a particular strain, following RNA degradation, the aspiration response of the treated DFC typically no longer exhibits saturation and instead continuously deforms under the applied pressure (Fig. 4*I* and Movie S9). This suggests that RNA degradation fluidizes the nucleolus.

We perform experiments to estimate the RNase-treated GC–DFC interfacial tension in the same manner as the previous section. The combined effect of the GC interface and the GC–DFC interface has an apparent interfacial tension of 8.0 ± 2.0 µN/m (*SI Appendix*, Fig. S10*C*). Under the assumption that RNase does not change the GC interfacial tension measured above (1.7 µN/m), we estimate that the interfacial tension at the RNase-treated GC–DFC interface is approximately 6.3 ± 2.0 µN/m, which is approximately ten times higher than the estimated upper bound for the untreated GC–DFC interfacial tension.

With this estimate of the total interfacial tension, we can estimate the total Laplace pressure and fit the treated DFC curves to a Newtonian model, as we did in the case of the fluid-like GC. We find an effective DFC viscosity of approximately 1,000 ± 400 Pa∙s. Thus, while treatment causes the DFC to behave more fluid-like, removing the effective elastic characteristics, it appears to result in a much higher effective viscosity of the material. The rapid coalescence of RNase-treated DFC therefore appears to be driven primarily by the increased interfacial tension. However, our estimated values alone are not enough to account for the two orders of magnitude decrease in the inverse capillary velocity, indicating that there may be either confounding factors in the geometry and flow of GC and treated DFC within the micropipette, or potential inaccuracies in estimating inverse capillary velocity through coalescence analysis.

We note that there are some cases where the RNase treatment does not completely remove the solid-like aspiration characteristic (Fig. 4*I*), which may indicate spatially varying material properties within the RNase-treated DFC. Additionally, in the case of the GC, precise measurements of properties under RNase-treated conditions are particularly challenging, since the thickness of the layer of GC surrounding the DFC is reduced (Fig. 4 *G* and *H*). Taken together, our analysis of RNA-dependent changes in DFC morphology, fusion dynamics, and aspiration responses following RNA degradation suggests that the viscoelastic solid-like properties of the DFC, and the DFC–GC interfacial tension, are both dependent on the presence of RNA.

## Discussion

**Fig. 5.**
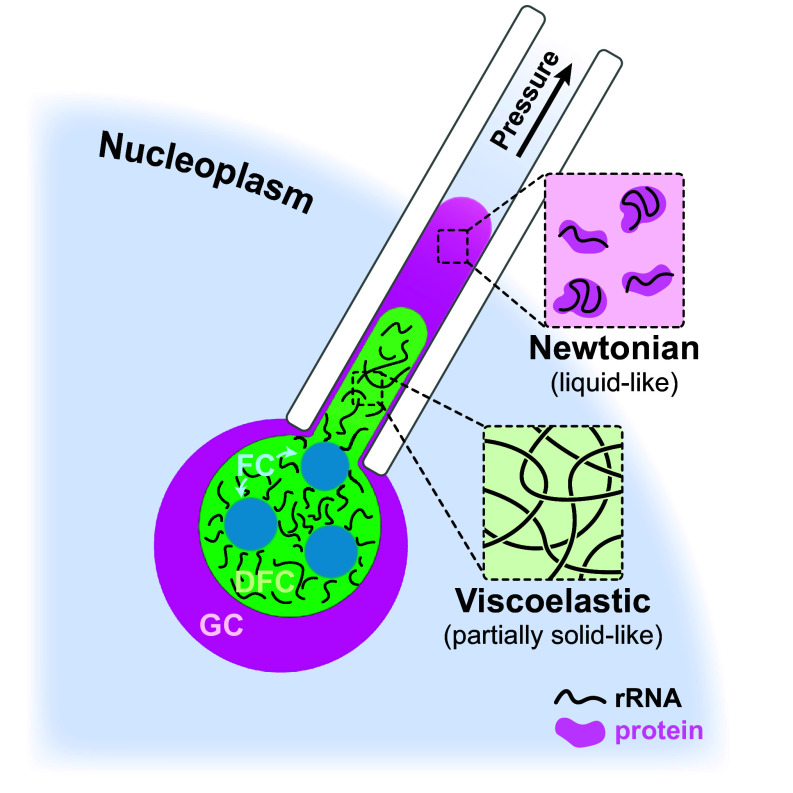
Schematic of aspirating the nucleolus into a micropipette. Within the DFC, the dense packing of nascent rRNAs appears to give rise to viscoelastic properties. The sequential cleavage, modification, folding, and assembly of these nascent rRNAs, as they migrate radially toward the nucleolar periphery, facilitates their compaction into preribosomal particles, which likely gives rise to the more fluid-like environment in the GC.

The nucleolus is the most transcriptionally active site in the genome, producing ~80% of the total RNAs inside cells and coordinating their assembly with proteins to form ribosomes (53, 54). However, until now the material properties of this RNA-rich environment have been difficult to probe directly due to technical limitations. Here, using MPA, we directly characterize the material properties of transcriptionally active *X. laevis* nucleoli in a near-native environment, and we show how these properties are RNA-dependent and spatially vary across the condensate. Specifically, our results suggest that the outermost nucleolar compartment, the GC, is a Newtonian-like fluid, while the inner compartment, the DFC, exhibits RNA-dependent viscoelastic properties (Fig. 5).

The characterization of nucleolar material properties described here represents significantly more direct measurements than those previously described. Commonly used techniques, like FRAP and fusion assays, require making a variety of assumptions, are prone to overinterpretation, and are sensitive to potential artifacts from the overexpression of fluorescent proteins. For example, in past work, estimation of GC viscosity required indirect measurements combining multiple different techniques (e.g., coalescence and sessile droplet shape) using nucleoli with overexpressed NPM1-RFP (44). Here, we use MPA to characterize the viscosity and Newtonian-like behaviors of the GC, as well as its interfacial tension. Similarly, we use MPA to characterize the effective RNA-dependent DFC viscoelasticity, and to estimate the impact of RNA on the DFC–GC interfacial tension, measurements which have not been reported before due to the technical limitations of previous techniques.

Our results demonstrate that RNA is a key contributor to nucleolar material properties, as its degradation clearly fluidizes the DFCs. This is consistent with prior studies suggesting that rRNA forms a gelled network within purified nucleolar protein (NPM1) condensates (55), and more broadly with various in vitro studies on the effect of RNA on condensate properties (56, 57). At the FC–DFC boundary, there is a high density of nascent rRNAs, as seen in early electron micrographs (58). As previously proposed (43), this close packing of several kilobase-long nascent rRNAs could promote physical entanglement and/or intermolecular hybridization, which would contribute to a partially solid-like DFC, consistent with our experimental observations (Fig. 3*B*). Our data also suggest that RNA not only has a notable effect on bulk viscoelasticity but also may markedly decrease the DFC–GC interfacial tension. The highly concentrated nascent rRNAs that are expected to underlie this effect may thus effectively behave like a surfactant, decreasing the otherwise relatively high DFC–GC interfacial tension, analogous to a previous suggestion on the effect of rRNA at the DFC–FC interface (59).

We note that there are several limitations of our work due to technical challenges that future studies could potentially overcome. While we observe Newtonian-like behaviors on timescales of ~10 s, this does not necessarily exclude the possibility of viscoelastic GC behavior on shorter timescales, which we do not observe due to spatial and temporal limitations of our system. For example, with faster imaging, future work could potentially perform cyclic MPA assays to examine the frequency-dependent viscoelastic properties of the nucleolus (60). Through careful modeling of the full three-dimensional flow problem, which would include accounting for the multiphasic nature of the nucleoli and small changes to the geometry of unaspirated portions of the DFCs during MPA measurements, future studies may be able to even more accurately quantify the material properties of the GC and DFC. Additionally, here we treat the DFC as a continuum material, but in reality, it contains an additional internal phase, the FC, which could potentially have different properties than the rest of the DFC. In several examples, such as in Fig. 2*G*, we observe that the onset of constant strain is coincident with the FC phase entering the pipette (visible in the images as the low FIB1 intensity puncta in the DFC). This is consistent with our model, as we expect that the regions closest to the FC–DFC boundary should have the highest concentration of entangled nascent rRNAs, and should thus be the most solid-like. Indeed, even within the “continuum DFC phase,” there is likely spatial variation in viscoelasticity, as rRNA becomes progressively more processed. Future studies could more closely examine the spatially varying RNA-dependent viscoelastic properties within the DFC, and how rRNA in the DFC could be fluidized through sequential cleavage, folding, and assembly. Finally, while the *X. laevis* oocyte provides a system with exceptionally large nuclear condensates, because the oocyte cytoplasm is opaque, it is necessary to isolate the GV from the cytoplasm for imaging. While the time since GV isolation does not appear to impact the relative shape of the GC and DFC aspiration curves, the viscosity of the GC is significantly affected (*SI Appendix*, Fig. S7*C*). This observation potentially hints at the dependence of GC viscosity on metabolic activity or ATP concentration (27, 49), intriguing possibilities for future exploration.

Our findings underscore the intimate links between the material properties of the nucleolus and its RNA components, which are at the heart of its function in ribosome biogenesis. The nucleolar viscosity will impact the timescales of RNA and protein mobility and thus the rate of ribosome production. It is possible that nucleolar viscosity is dynamically tuned to optimize ribosome biogenesis given the environmental conditions, much like how cells can modulate their cytoplasmic viscosity in response to temperature and energy perturbations (61, 62). Furthermore, an elastic RNA network within the DFC could act to kinetically trap unprocessed nascent rRNAs (43). Limiting the diffusivity of these nascent rRNAs may be advantageous for enforcing the complex sequential nature of ribosome maturation; only once rRNAs are correctly processed can they be released from the gel and gain access to a new set of processing and assembly factors. Recent work has indeed begun to suggest that perturbation of nucleolar material properties can lead to defects in ribosome biogenesis (4).

Cancer, aging, and ribosomopathies are also all associated with defective ribosome production and striking changes in nucleolar morphology, which could reflect aberrant material properties (63). By adapting MPA to study nucleoli, our study opens many possibilities for exploring the biological impact of nucleolar material properties. These findings also pave the way for studies on the relationship between material properties and biological function in other biomolecular condensates, including the readily accessible large Cajal bodies within *X. laevis* oocytes, and potentially other model systems.

## Methods

### *X. laevis* Oocyte Preparation.

Ovaries were collected from healthy adult female *X. laevis* frogs that were euthanized by immersion in 1 to 3 g/L ethyl 3-aminobenzoate methanesulfonate (Sigma-Aldrich), followed by decapitation, under Princeton University Institutional Animal Care and Use Committee (IACUC) protocol No. 2070. The ovaries were incubated in OR2 saline (64) supplemented with 1% streptomycin and penicillin (Gibco). To remove the follicular layer, the oocytes were mechanically separated with forceps under a Nikon SMZ745 stereo microscope and incubated in 2 mg/ml collagenase (Sigma-Aldrich) dissolved in OR2 with slow rotation for 80 min. The isolated Stage V-VI (1 to 1.3 mm) oocytes were stored and used in all experiments at room temperature (~22 °C) in OR2 for up to one week following isolation.

### DNA Constructs and mRNA Synthesis.

FIB1-eGFP and NPM1-RFP constructs on a pCS2+ backbone were used as previously described (27). The plasmids were linearized by NotI (New England Biolabs (NEB)) restriction enzyme digestion. The linearized plasmids were purified with the QIAquick Gel Extraction (Qiagen) and DNA Clean & Concentrator-5 kits (Zymo Research), and used as a template for in vitro transcription using the HiScribe SP6 RNA Synthesis Kit (NEB). To produce capped RNAs, 3´-O-Me-m7G(5’)ppp(5’)G RNA Cap Structure Analog (NEB) was added to the transcription reaction at a ratio of cap:GTP = 4:1. The DNA template was digested by DNase I treatment, and the synthesized mRNA was purified using RNA Clean & Concentrator-25 columns (Zymo) and stored at −80 °C in nuclease-free water.

### Oocyte Microinjection.

The needles used for microinjections were pulled from glass capillaries with filament (World Precision Instruments (WPI)) using a P-97 Flaming/Brown type micropipette puller (Sutter Instrument). The needles were backfilled with the injection solutions by capillary action, connected to a FemtoJet microinjection pump (Eppendorf), and mounted on a mechanical micromanipulator (Narishige). The needle tip was broken to the desired opening diameter with forceps. The injection volume was calibrated by using a stage micrometer to measure the size of droplets ejected into a dish of mineral oil under a specified injection pressure and time. To fluorescently label the nucleolar subcompartments, oocytes were microinjected cytoplasmically with FIB1-eGFP and NPM1-RFP mRNAs dissolved in nuclease-free water (~2.5 ng mRNA/oocyte) under a Nikon SMZ745 stereo microscope and incubated overnight.

### Microscopy.

To image the oocyte nucleus (GV), the GV was first extracted under mineral oil as previously described (49), then transferred to a mineral oil-filled 35 mm glass bottom dish (MatTek). The subsequent imaging experiments were completed within two hours of GV isolation. Unless specified below, all images were taken using a Nikon A1 point-scanning confocal microscope equipped with 20X air (Plan APO, NA 0.75) and 60X oil-immersion (APO, NA 1.40) objectives, 488, 561, and 640 nm lasers, and a diascopic detector for simultaneous capture of fluorescent and differential interference contrast (DIC) microscopy images.

Fluorescently labeled 5-Ethynyl Uridine (5-EU) containing RNAs in the nucleolus (*SI Appendix*, Fig. S1*B*) were imaged using a CSU-X1 spinning disk (Yokogawa) on an inverted microscope Eclipse Ti body (Nikon), equipped with a 100x oil immersion objective (Apo TIRF, NA 1.49) and 488, 561, and 640 nm lasers. The aspiration of silicone oil droplets (Fig. 1 *E* and *F* and *SI Appendix*, Fig. S2*C*) was imaged on a Ti2-A inverted widefield fluorescence microscope (Nikon) with a 60x water immersion objective (Plan Apo VC, NA 1.2).

### Micropipette aspiration.

Micropipettes were pulled from glass capillaries with no filament (WPI) using the P-97 Flaming/Brown type micropipette puller (Sutter Instrument). The micropipette tips were then cut to an opening diameter ~1 to 2 µm and bent to ~45° using a MF200 Microforge (WPI). To reduce clogging from the GV membrane and surrounding mineral oil, the tips of the micropipettes were coated overnight in 1% (w/v) Pluronic F-127 (PF-127) (Sigma-Aldrich). The PF-127 solution was ejected and the micropipette tip was then washed 2 to 3 times with water by applying pressures with an air-filled syringe. Prepared micropipettes were filled with basic oocyte nucleus solution (65) using MicroFil Needles (WPI). The buffer-filled micropipette was attached to a needle holder (Tritech Research) and connected to a water reservoir through tubing. To control the aspiration pressure, the water column height was adjusted using connected 20 ml, 60 ml, and 150 ml syringes. To control the micropipette position and angle, a motorized 5171 micromanipulator (Eppendorf) was mounted on the Nikon A1 point-scanning confocal microscope described above with a custom machined adapter.

The GV was isolated into mineral oil in 35 mm glass bottom dishes precoated with Poly-D-lysine (MatTek) to improve adhesion of the GV membrane to the glass. The micropipette tip was adjusted to be parallel to the dish surface, and inserted into the GV under high ejection pressures (~1 kPa) to prevent clogging. The pressure was then immediately adjusted to approximately zero. In a successful insertion, the tip of the micropipette had no debris that partially blocked the opening. Damaged GVs (typically with visible flow of residue yolk granules into the nucleoplasm around the micropipette entry site) were discarded.

To calibrate the zero pressure of the setup, we took advantage of the fact that the leading portion of the GC occasionally fragments inside the pipette under suction pressure, forming a small, freely slipping slug (*SI Appendix*, Fig. S2*D*). An ejection pressure was next applied to remove the unfragmented portion of the nucleolus so that only the small slug remained inside the pipette. The pressure was then adjusted in 2.5 Pa increments until the slug only moved inside the pipette under Brownian motion – we define this as the zero pressure.

The deformation of nucleoli labeled with both NPM1-RFP and FIB1-eGFP into micropipettes under calibrated aspiration pressures was recorded at an imaging frequency of 2 s/frame under 488 nm and 561 nm lasers with simultaneous DIC images. The deformation of nucleoli with only one fluorescently expressed protein was recorded at 1 s/frame. The pipette and the nucleolar–nucleoplasm interface (both inside and outside the pipette) were clearly visible in the DIC images.

To analyze the MPA videos, we only included data where there are no signs of necking instabilities, which makes the aspiration response more complex and difficult to interpret (*SI Appendix*, Fig. S8). Poisson shot noise was first removed using Nikon’s NIS-Elements Denoise.ai software (66). To correct for pipette drift, the videos were registered using the FIJI plugin HyperStackReg (Version 5.7), translation transformation (67, 68). FIJI was used to generate a kymograph of the RFP and eGFP fluorescence intensity in the center of the pipette (“Reslice” function), using the DIC channel to identify the pipette position. The kymograph was segmented based on fluorescence intensity in ImageJ. A MATLAB (R2023a) script was then used to measure the length of each subcompartment inside the pipette (*L*_p_) over time using the kymographs. DIC images of the pipette were used to measure the radius of the pipette (*R*_p_) using FIJI.

To more clearly visualize the nucleoplasm inside the micropipette, in some experiments (*SI Appendix*, Fig. S2 *A* and *B*), the nucleoplasm was fluorescently labeled by injecting oil-isolated GVs with ~0.3 nl 20 mg/ml 150 kDa FITC-dextran (Sigma-Aldrich) (~1% total GV volume).

Aspiration of N18000 viscosity standard silicone oil droplets (Cannon Instrument Company) was performed using a previously described setup (36). To achieve nonwetting conditions, both the micropipette and glass bottom dish (Cellvis) were filled with 100% ethanol before aspiration. The filled pipette was mounted onto the micromanipulator (Scientifica) and the pipette tip was moved into the oil droplet. A 0.2 s/frame video was recorded to track the deformation of silicone oil. The analysis of the data follows the previously published protocol (36).

### RNA Degradation.

To degrade RNAs, isolated GVs were injected with ~0.3 nl of 10 mg/ml RNase A in 50 mM Tris-HCl (pH 7.5) and 50% (v/v) glycerol (Thermo Fisher). The nucleoli were imaged starting ~10 min following injection.

### Nucleolar Fusion Experiments.

As previously described (44), oocytes expressing NPM1-RFP and FIB1-eGFP were incubated with 2 µg/ml Latrunculin A (Abcam) for 1 to 2 h while gently rotating. Following treatment, the GV was isolated into an oil-filled 35 mm glass bottom dish (MatTek) and immediately imaged using a Nikon A1 point-scanning confocal microscope every 30 s. For RNase A fusion experiments, the isolated actin-disrupted GV was injected with ~0.3 nl 10 mg/ml RNase A before imaging. For RNase-A treated nucleoli, due to the fast fusion dynamics, the nucleoli were imaged every 4 s.

The aspect ratio of fusing nucleoli was determined by segmenting the nucleoli based on fluorescence intensity, fitting the nucleoli to an ellipse in FIJI, and then dividing the major axis length by the minor axis length. The aspect ratios of relaxing nucleoli were fit to the equation $AR=1+\left({\mathrm{AR}}_{0}-1\right)e^{-t/\tau }$. The length scale l is the geometric mean of the major and minor axes at t = 0. We find inverse capillary velocity η/γ by fitting the data to the equation τ=(η/γ)·l.

### 5-EU Labeling in Oocytes.

Oocytes expressing FIB1-eGFP and NPM1-RFP were injected cytoplasmically with 50 nl of 9 mM 5-EU dissolved in water (Thermo Fisher) and then incubated at room temperature for the pulse length. To prepare the nucleoli for fixation and click reaction steps, we followed an established protocol for examining *X. laevis* nuclear contents (69). Specifically, the oocyte GV was dissected in 5:1 isolation media, and then immediately transferred to dispersal media. A pair of forceps was then used to break open the nuclear envelope, and the nuclear contents were centrifuged onto Poly-D-lysine coated glass coverslips at 4,000 rpm for 40 min. The nucleoli were then fixed for 15 min with 2% PFA in DPBS, followed by two washes with DPBS. To conjugate Alexa-647 (Alexa Fluor 647 Azide, Triethylammonium Salt, Thermo Fisher) to the incorporated 5-EU, we used the Click-iT RNA imaging kit (Thermo Fisher) according to manufacturer instructions. Imaging of fixed GV spreads was performed using a Nikon X1 spinning disk microscope with a 100X oil-immersion objective described above. Similarly, oil-isolated GVs were injected with 1.2 nl of 100 mM 5-EU dissolved in water, incubated at room temperature for the pulse length, transferred to dispersal media, then treated as described above. Imaging was performed using the Nikon A1 point-scanning microscope with a 60X objective.

### Calculation of Material Parameters and Uncertainties.

To find the interfacial tension of GC and RNase treated DFC, we conducted aspiration experiments for a range of applied pressures, generating several traces of $L_{p}$ versus t. We then fit these traces to Eq. **1**, with A=C=0. We generated at least three values of the fitting parameter B as a function of the applied pressure, as well as associated SE of regression for each of the fits. We performed a linear fit between B and the applied pressure, using the SE from the first fit as weights in a weighted least squares algorithm. This was accomplished with Scipy’s curve_fit function with sigma set as the SE and setting absolute_sigma to true. The intercept of the resulting fit can be interpreted as the Laplace pressure acting on the system due to the various interfaces in the pipette. For each sample, we converted this to an interfacial tension measurement using the radii of the pipette and the sample. Finally, we obtained the reported value by taking an average over the samples.

To obtain the reported material parameters, we proceeded by fitting Eq. **1** to each dataset as described in the text, resulting in a set of fitting coefficients. For nuclei where multiple traces are collected, we averaged the coefficients to avoid overweighting on samples with more trials. We used the reported value of the interfacial tension along with the pipette radii to calculate the effective Laplace pressure of each sample. We then used the Laplace pressure, the known applied pressure, and the value of the fitting coefficients to calculate the material parameters following the formula described in *SI Appendix*, Table S1. We again took an average over the samples to produce the reported values found in the text.

We report uncertainties on material properties as one SEM, as all material properties are averaged over several individual nucleoli. While the SEM does not account for the measurement uncertainty (0.2 µm on length measurements and 1 Pa on pressure measurements), our samples exhibited large variability relative to each other, resulting in sample variances much higher than the variance obtained by propagation of measurement uncertainty. Thus, we chose to report the SEM as the most conservative estimate. The only measurement this did not hold true for was the viscosity measurement of GC nucleoli less than one hour old, which we report as 38 ± 10 Pa∙s. Were we instead to compute the uncertainty using standard propagation of error formula, we would find 38 ± 12 Pa∙s. The data we used to compute the estimates of material parameters, code used for analysis, and estimates of the parameters with uncertainties calculated as both the SE and with propagation of error may be found at (70).

## Supplementary Material

Appendix 01 (PDF)

Movie S1. Aspiration of the GC into a pipette, as in Fig. 1G. The nucleolar subcompartments are fluorescently labeled (GC in magenta, DFC in green). Time = min:s. Scale bar: 5 μm.

Movie S2. Aspiration of the nucleolus at constant pressure, as in Fig. 2A. Time = min:s. Scale bar: 5 μm.

Movie S3. Relaxation of the GC following ejection from the pipette, as in Fig. 2C. Time = min:s. Time = 0 corresponds to when the nucleolus leaves the pipette. Scale bar: 5 μm.

Movie S4. Relaxation of the DFC following ejection from the pipette, as in Fig. 2D. Time = min:s. Time = 0 corresponds to when the nucleolus leaves the pipette. Scale bar: 5 μm.

Movie S5. Aspiration of the DFC, as in Fig. 2G. Time = min:s. Scale bar: 5 μm.

Movie S6. Coalescence of untreated DFCs following actin network disruption by Latrunculin-A, as in Fig. 4D. Time = hr:min:s. Scale bar = 20 μm.

Movie S7. Coalescence of RNase A treated DFCs following actin network disruption by Latrunculin-A, as in Fig. 4D. Time = min:s. Scale bar = 20 μm.

Movie S8. Relaxation of an RNase-treated nucleolus, as in Fig. 4G. Scale bar: 3 μm.

Movie S9. Aspiration of an RNase-treated nucleolus, as in Fig. 4H. Scale bar: 3 μm.

## Data Availability

Data and analysis code data have been deposited in cheng_et_al_2025_data (https://github.com/SoftLivingMatter/cheng_et_al_2025_data) (70). All study data are included in the article and/or supporting information.
