# Supplementary material for: Micropipette aspiration reveals differential RNA-dependent viscoelasticity of nucleolar subcompartments
Source: Proc Natl Acad Sci U S A. 2025 May 28;122(22):e2407423122. doi: 10.1073/pnas.2407423122 (PMC12146704; doi:10.1073/pnas.2407423122)
Supplement: Supplementary file 1 — Appendix 01 (PDF) [file pnas.2407423122.sapp.pdf]

PNAS

Supporting Information for

Micropipette aspiration reveals differential RNA-dependent viscoelasticity of nucleolar subcompartments

Holly H. Cheng[1], James V. Roggeveen[2], Huan Wang[3], Howard A. Stone[2,6*], Zheng Shi[3*], Clifford P. Brangwynne[4,5,6,7*]

1. Department of Molecular Biology, Princeton University, Princeton, NJ 08544, USA
2. Department of Mechanical and Aerospace Engineering, Princeton, NJ 08544, USA
3. Department of Chemistry and Chemical Biology, Rutgers University, Piscataway, NJ 08854, USA
4. Department of Chemical and Biological Engineering, Princeton University, Princeton, NJ 08544, USA
5. Omenn-Darling Bioengineering Institute, Princeton University, Princeton NJ, 08544, USA
6. Princeton Materials Institute, Princeton University, Princeton, NJ 08544, USA
7. Howard Hughes Medical Institute, Chevy Chase, MD 21044, USA

*Correspondence: cbrangwy@princeton.edu; zheng.shi@rutgers.edu; has@princeton.edu
Lead contact: cbrangwy@princeton.edu

This PDF file includes:

Supplementary Discussion
Figures S1 to S11
Tables S1
Legends for Movies S1 to S9
SI References

Other supporting materials for this manuscript include the following:

Movies S1 to S9

Supplementary Discussion

Analysis of micropipette aspiration curves

For fitting our GC data, using the general model (equation 1) did not converge to a result using 8000 evaluations of Scipy's curve fitting function. We thus constrained the fit by setting $C = D = 0$. When we allowed for nonzero values for A, we found that interpreting this fitting parameter in terms of a Maxwell model would correspond to a high elastic modulus, given that the data is normalized such that the initial strain is 0. This elastic modulus is unphysical and results from the fact that fitting a Maxwell model to a Newtonian fluid requires taking $E \to \infty$. Our data does not provide evidence of a Maxwell-like behavior, so we interpret the data as Newtonian. As such, we can set $A = 0$ given that the initial strain is 0 and refit B to find $B = 0.06$. This is consistent with a material response of a Newtonian-like fluid, with strain increasing as a linear function of time.

While it is difficult to accurately measure the DFC-GC interfacial tension because it is always surrounded by a thin film of the GC, we observed that the DFC flows into the pipette at 5 Pa. Given that the Laplace pressure of the GC-nucleoplasm interface is, on average, 3.4 Pa, the upper bound of the DFC Laplace pressure is ~1.6 Pa. The average Laplace pressure was determined by taking the reported value of the GC interfacial tension of 1.7 µN/m, computing the Laplace pressure for each pipette used in our experiments, and then taking the mean. For the typical pipette radius of ~0.5 µm and a DFC radius of ~3 µm, this corresponds to an interfacial tension of around 0.5 µN/m. The contributions from DFC interfacial tension to the aspiration dynamics is thus small in comparison to the applied 20 Pa during the measurements. Consistent with the prediction of small DFC-GC interfacial tension, by combining our measurements of DFC viscosity by MPA (~250 Pa·s) with DFC inverse capillary velocity from fusion assays (~300 s/µm), we can estimate the DFC-GC interfacial tension to be approximately 0.8 µN/m. The expectation of small DFC-GC interfacial tensions justifies ignoring their relative contribution to the aspiration dynamics, and in any case, should not change the relative shape of aspiration curves for GC versus DFC.

In addition to aspiration data where the material is drawn into the pipette, we can also analyze relaxation data, where the material is expelled from the pipette. While analysis of the relaxation segments would allow us to extract an estimate for the interfacial tension (51), the small Laplace pressure generated by the weak interfacial tension of *X. laevis* nucleoli leads to very slow relaxation.

Compounding these issues, we found that the relaxation data were poorly fit by the viscoelastic models. In the case of the DFC, as the interfacial tension effects are relatively weak, the strain evolves very slowly compared to the aspiration case. Under such conditions, the leading-order behavior of the solid models looks linear and would be indistinguishable from a Newtonian fluid. When we analyzed the segments, fitting the Kelvin-Voigt solid-like model was numerically unstable and did not produce reasonable measurements. The only fit that was stable was a simple linear fit, consistent with measuring the period of an exponential relaxation. A further issue is that in the case of relaxation the material wants to completely relax out of the pipette, and thus even a solid-like material with a terminal relaxation to a defined strain will never achieve a measurable steady-state strain under these conditions. These factors led to challenges achieving numerical convergence when trying to fit the data traces to the theoretical models.

Analysis of the GC relaxation data showed a good fit with the Newtonian model assumed under aspiration conditions. There was some evidence in a few experimental traces of behavior consistent with a third-order model (Jeffery or Oldroyd-B), which would cause the data to curve at early times. However, this effect was not robust enough to fit across all available datasets so we focused only on two-element models for simplicity.

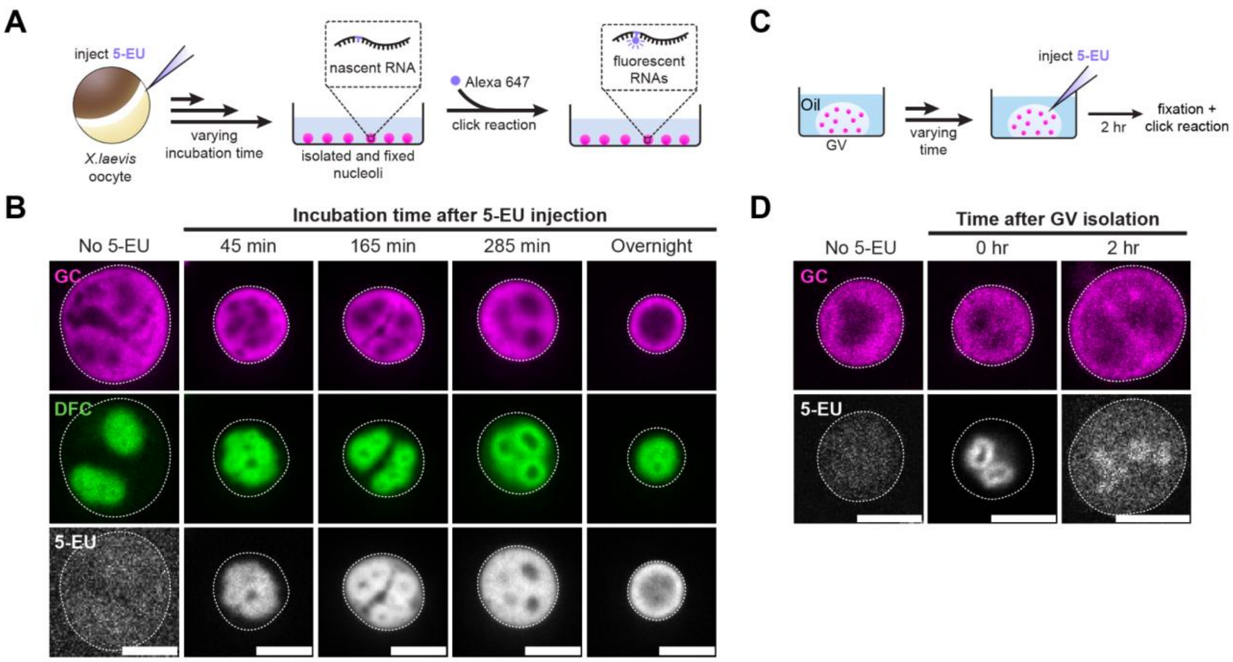

Figure S1: Stage V-VI *X. laevis* oocyte nucleoli are transcriptionally active.
(A) Schematic of a 5-EU-based protocol for labeling nascent RNA production in *X. laevis* oocytes.
(B) Representative images from the experiment shown in panel A are arranged in increasing time between the injection of 5-EU and fixation. The GC and DFC are fluorescently labeled by NPM1-RFP and FIB1-eGFP, respectively. 5-EU containing RNAs are conjugated to Alexa Fluor 647. The dashed lines indicate the nucleolar boundary. Scale bar: 5 µm.
(C) Schematic of protocol for assessing transcriptional activity in the nucleolus after GV isolation.
(D) Representative images of nucleoli from the experiment shown in panel C. The low intensity regions in the GC show the location of the DFC/FCs. The 5-EU images for No 5-EU, 2 hr oil-isolation were captured using the same imaging settings (laser power and gain parameters) and are displayed with the pixel intensities scaled to the same minimum and maximum values. The 5-EU image for 0 hr oil-isolation was taken at lower laser power due to saturation. The dashed lines indicate the boundaries of the nucleoli. Scale bar: 5 µm.

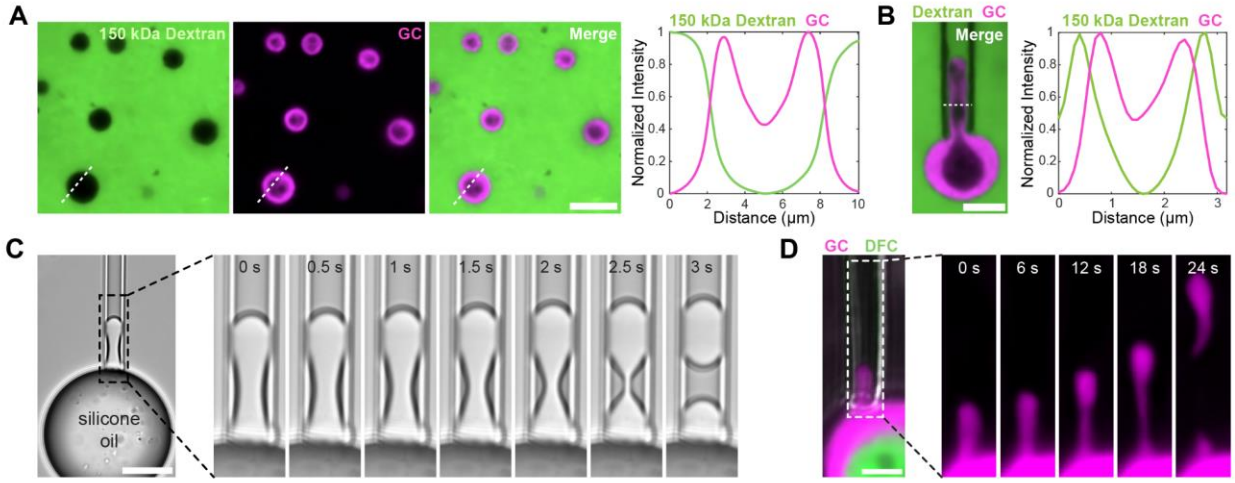

Figure S2: Nucleoli do not wet the inner wall of the micropipette.

(A) The nucleoplasm was labeled by injecting oocyte nuclei with 150 kDa FITC-dextran (green), which is excluded from the nucleoli. Nucleoli were labeled by expressing the GC protein NPM1-RFP (magenta). The fluorescence intensities of FITC-dextran and NPM1-RFP along the white line are plotted on the right. Scale bar: 5 µm.

(B) The fluorescence intensities of FITC-dextran and NPM1-RFP along the white line inside the pipette are plotted on the right, showing a film of nucleoplasm between the GC and pipette wall. Scale bar: 3 µm.

(C) Example of oil fission inside a micropipette. Scale bar: 15 µm.

(D) Example of GC fission. Scale bar: 3 µm.

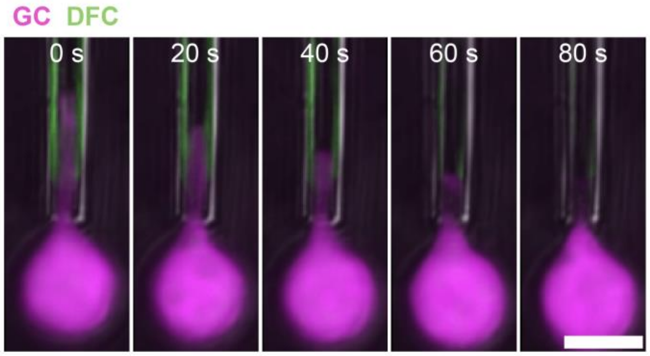

Figure S3: Ejected GC (with no enclosed DFC) quickly becomes spherical as it exits the pipette. Scale bar: 5 µm.

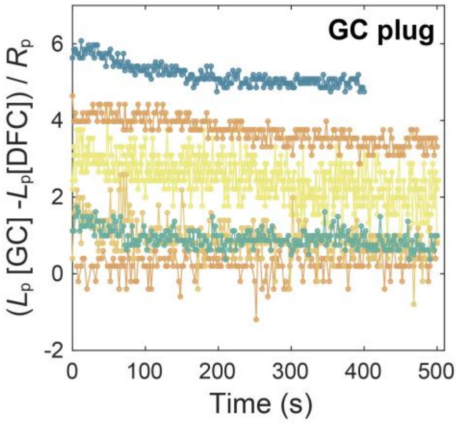

Figure S4: Length of the GC plug during the DFC aspiration measurements ((L_p[GC] - L_p[DFC]) / R_p). Only measurements where both the GC and DFC were fluorescently labeled are included.

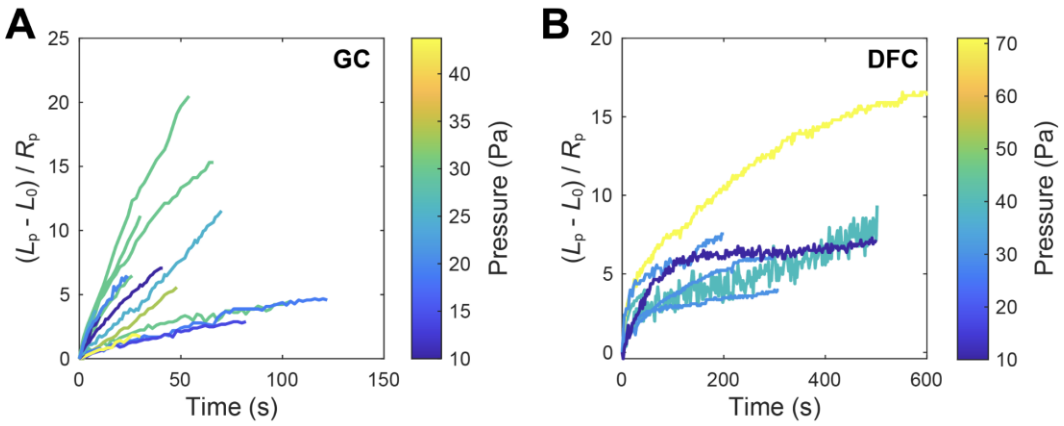

Figure S5: Aspiration of the nucleolar subcompartments of the DFC and GC at different pressures.
(A) Aspiration responses of the GC at other pressures not shown in Figure 3.
(B) Aspiration responses of the DFC at other pressures not shown in Figure 3.

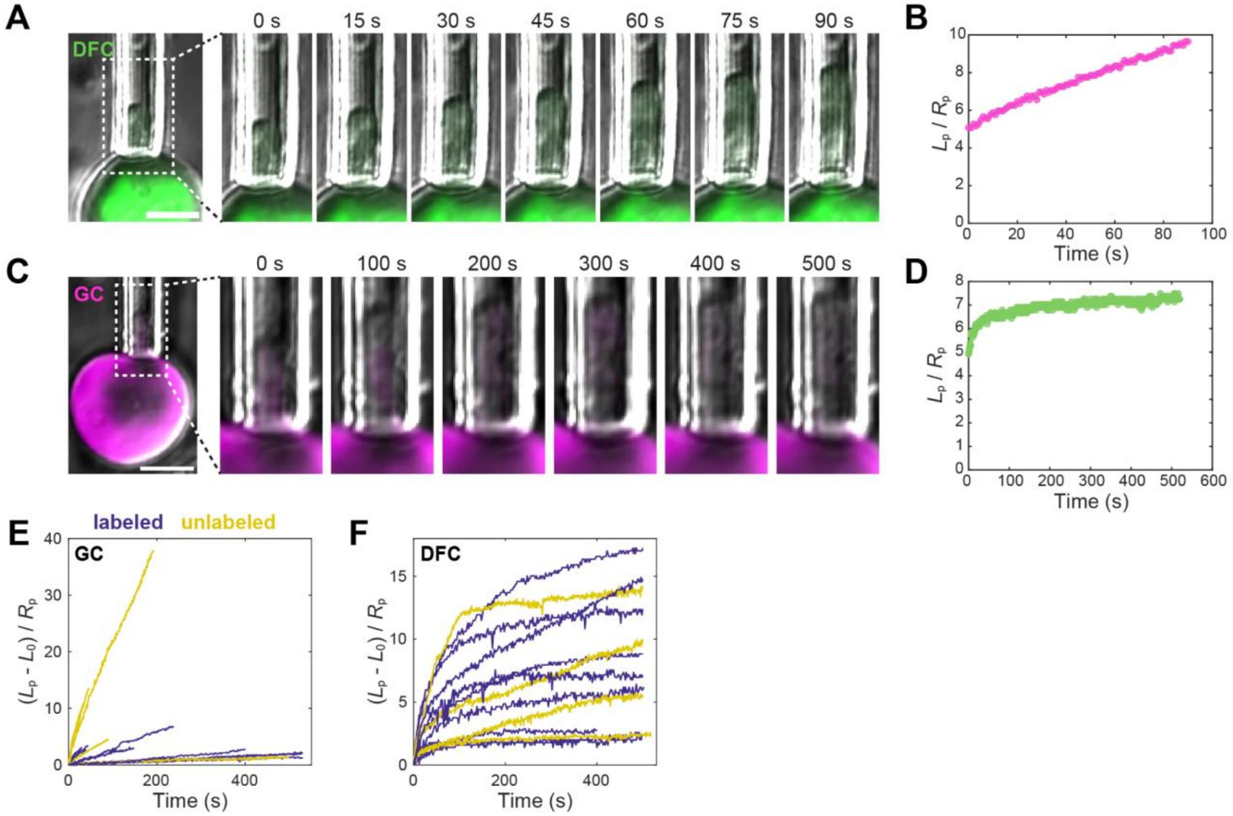

Figure S6: Shape of aspiration responses of the GC and DFC are independent of fluorescent protein expression.

(A) Aspiration of GC without NPM1-RFP expression. The GC-nucleoplasm interface and pipette are visible in the DIC image overlays. Overlay of DIC images with FIB1-eGFP (DFC) fluorescence. Scale bar: 5 µm.

(B) Quantification of GC response shown in panel A.

(C) Aspiration of DFC without FIB1-eGFP expression. Scale bar: 5 µm.

(D) Quantification of DFC response shown in panel C. Because the length of the GC plug ahead of the DFC inside the pipette remains approximately constant (Fig. S5*C*), the deformation of the DFC can be inferred from the length of the GC inside the pipette.

(E) Comparison of the response of the GC in the presence (purple) and absence (orange) of NPM1-RFP expression, corresponds to the data shown in Fig. 3*A*.

(F) Comparison of the response of the DFC in the presence (purple) and absence (orange) of FIB1-eGFP expression, corresponds to the data shown in Fig. 3*B*.

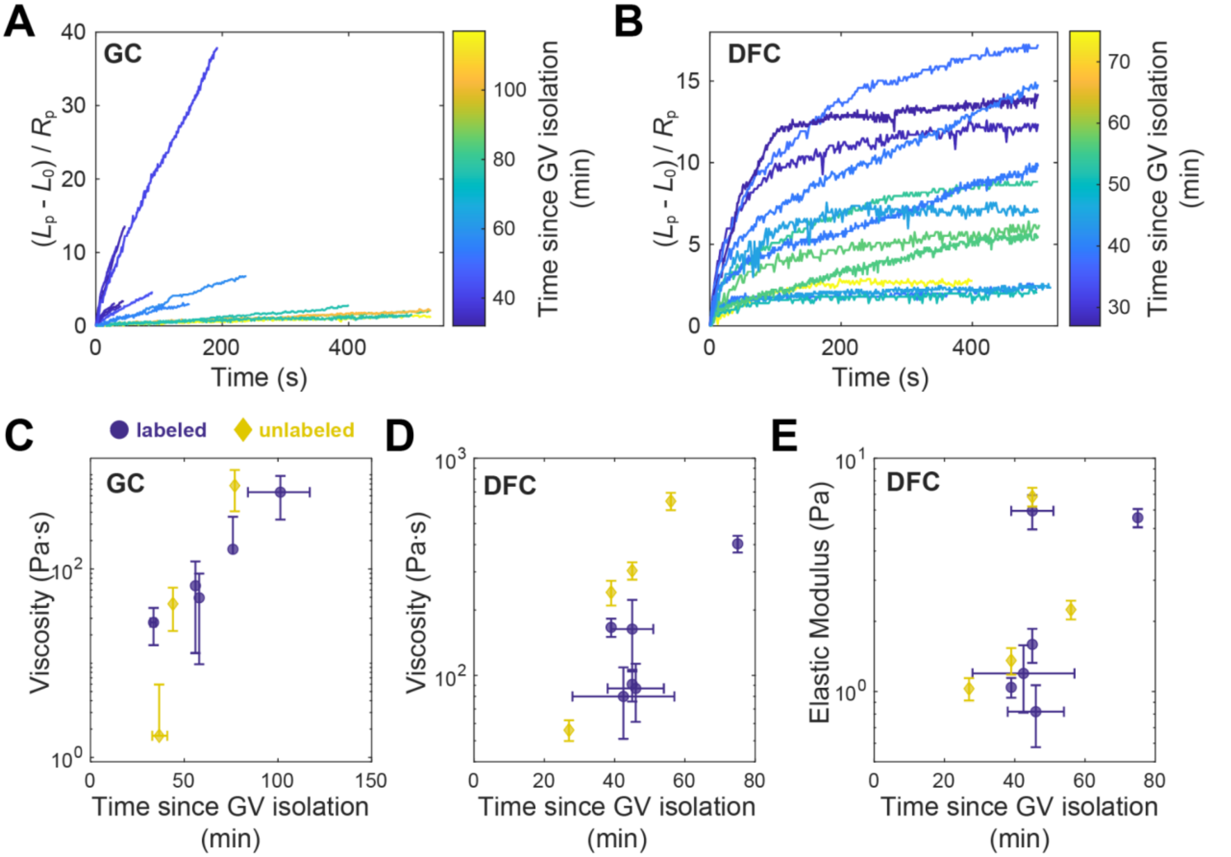

Figure S7: Correlation between nucleolar subcompartment material properties and the time since the GV was isolated from the oocyte.
(A-B) Aspiration response of the GC at 5 Pa (A) and DFC at 20 Pa (B) colored by time since the GV was isolated from the oocyte into mineral oil. Data corresponds to Figure 3.
(C) Scatterplot of the relationship between the time since GV isolation and apparent GC viscosity. Each point represents a different sample. The vertical error bars show one standard error of the mean in cases where multiple measurements of a single sample were pooled and standard error of regression when only one measurement of a given sample was taken was taken. The horizontal error bars show the range of times the sample was measured at. Purple circles: expressing NPM1-RFP. Yellow diamonds: no NPM1-RFP expression. For the effect of time since isolation on viscosity, Spearman's rank correlation $\rho = 0.929$, $p = 0.002$. For the effect of labeling on viscosity, by two-sided Wilcoxon rank sum test, $p = 0.786$.
(D) Relationship between time since GV isolation and apparent DFC viscosity. For the effect of time since isolation on viscosity, Spearman's rank correlation $\rho = 0.554$, $p = 0.097$. For the effect of labeling on viscosity, by two-sided Wilcoxon rank sum test, $p = 0.476$.
(E) Relationship between time since GV isolation and apparent DFC elastic modulus. For the effect of time since isolation on elastic modulus, Spearman's rank correlation $\rho = 0.449$, $p = 0.193$. For the effect of labeling on elastic modulus, by two-sided Wilcoxon rank sum test, $p = 0.762$.

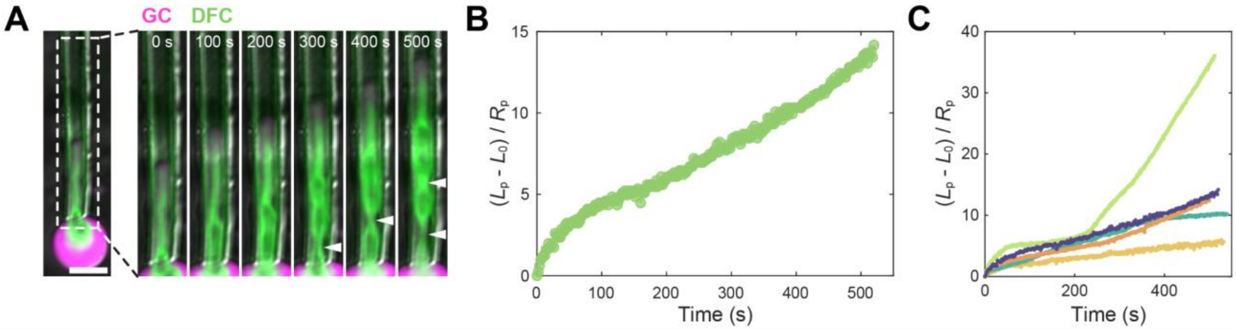

Figure S8: Examples of necking transitions of the DFC during aspiration.
(A) Timelapse images of a DFC aspiration (P = 20 Pa) with a necking transition. The white arrowheads indicate the positions of necking instabilities. Scale bar: 5 µm.
(B) Quantification of the position of the leading edge of the DFC inside the pipette shown in panel A.
(C) Additional examples (n = 5) of DFC measurements where necking instabilities occurred during aspiration at 20 Pa.

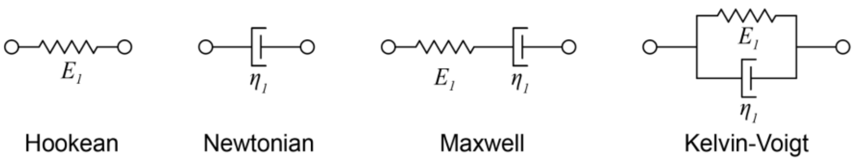

Figure S9: One- and two-component spring damper models. Maxwell and Kelvin-Voigt are considered viscoelastic models due to the combination of viscous and elastic components. Maxwell materials are fluid-like due to a long-time viscous response, while Kelvin-Voigt materials are solid-like due to a long-time elastic response.

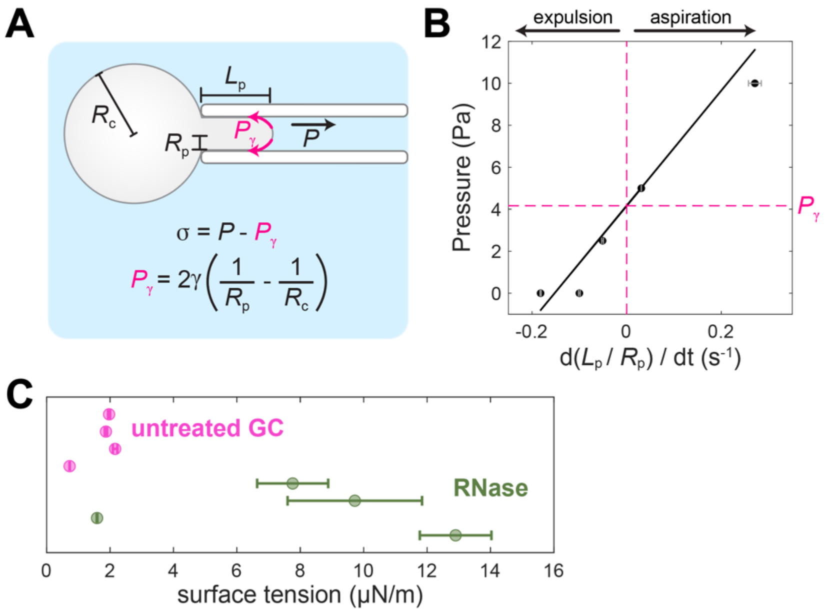

Figure S10: Approximation of nucleolar interfacial tension.
(A) Schematic and key equations describing the effects of interfacial tension on stress σ during MPA. Abbreviations: P = applied pressure, P_γ = Laplace pressure (in magenta), γ = interfacial tension, R_p = radius of the pipette opening, and R_c = radius of the droplet.
(B) Example plot of the applied pressure vs the aspiration rate $d(L_p / R_p) / dt$ for a GC aspiration. The black dots indicate the experimental data, the black line shows the linear fit. The y-intercept, shown by the intersection of the two dashed magenta lines, indicates the Laplace pressure P_γ is 4.2 ± 0.2 Pa in this example. The line of best fit is found using weighted least squares, which places less weight on the contribution of the sample at 10 Pa due to the relatively higher variance in the measured shear rate. The error bars represent one standard error of regression, computed from the covariance matrix returned by the curve fitting routine. Note that using standard least-squares for fitting, as we have done, underestimates the measurement error due to the fact that it does not account for uncertainty in the independent variable (applied pressure). However, we calculate the uncertainty of the parameters using the standard error of the mean over several samples, so the underestimate of the measurement uncertainty does not affect the final values of the reported error in condensate material properties, consistent with the discussion in Methods: Calculation of material parameters and uncertainties.
(C) Distribution of untreated GC/nucleoplasm interfacial tension in magenta and RNase-treated nucleolus apparent interfacial tensions (combined effect of the GC-nucleoplasm and GC-DFC interfaces) in green. The error bars represent one standard error.

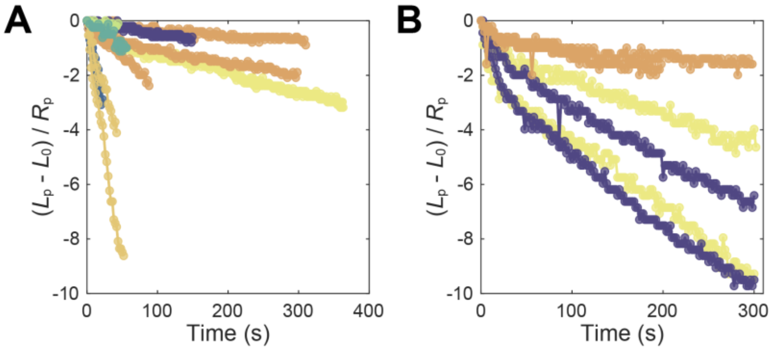

Figure S11: Relaxation of the GC and DFC upon release of suction pressure.
(A - B) Example response curves the GC (A) and DFC (B) when suction pressure is released (0 Pa). The dots represent the experimental data, with each color representing measurements from different nucleoli.

Table S1: Definitions of each fitting parameter in equation 1 for each of the four one- and two-component spring-damper models.

	A	B [1/T]	C	D [1/T]
Newtonian	ε_0	σ/η_1	0	0
Hookean	σ/E_1	0	0	0
Maxwell	$\varepsilon_0 + \sigma/E_1$	σ/η_1	0	0
Kelvin-Voigt	$\varepsilon_0 + \sigma/E_1$	0	-σ/E_1	E_1/η_1

These fitting parameters are defined in terms of the applied pressure σ, the initial strain ε_0 which we normalize to zero, and the strengths of the spring and dampers, E_1 and η_1 respectively. Schematics of each model are presented in Fig. S9.

Movie S1: Aspiration of the GC into a pipette, as in Fig. 1G. The nucleolar subcompartments are fluorescently labeled (GC in magenta, DFC in green). Time = min:s. Scale bar: 5 µm.
Movie S2: Aspiration of the nucleolus at constant pressure, as in Fig. 2A. Time = min:s. Scale bar: 5 µm.
Movie S3: Relaxation of the GC following ejection from the pipette, as in Fig. 2C. Time = min:s. Time = 0 corresponds to when the nucleolus leaves the pipette. Scale bar: 5 µm.
Movie S4: Relaxation of the DFC following ejection from the pipette, as in Fig. 2D. Time = min:s. Time = 0 corresponds to when the nucleolus leaves the pipette. Scale bar: 5 µm.
Movie S5: Aspiration of the DFC, as in Fig. 2G. Time = min:s. Scale bar: 5 µm.
Movie S6: Coalescence of untreated DFCs following actin network disruption by Latrunculin-A, as in Fig. 4D. Time = hr:min:s. Scale bar = 20 µm.
Movie S7: Coalescence of RNase A treated DFCs following actin network disruption by Latrunculin-A, as in Fig. 4D. Time = min:s. Scale bar = 20 µm.
Movie S8: Relaxation of an RNase-treated nucleolus, as in Fig. 4G. Scale bar: 3 µm.
Movie S9: Aspiration of an RNase-treated nucleolus, as in Fig. 4H. Scale bar: 3 µm.

SI References

1. K. Guevorkian, M.-J. Colbert, M. Durth, S. Dufour, F. Brochard-Wyart, Aspiration of biological viscoelastic drops. *Phys. Rev. Lett.* **104**, 218101 (2010).
